# Antibody-independent surface plasmon resonance assays for influenza vaccine quality control

**DOI:** 10.1007/s00253-024-13145-y

**Published:** 2024-04-24

**Authors:** Benjamin Serafin, Amine Kamen, Gregory de Crescenzo, Olivier Henry

**Affiliations:** 1 Department of Chemical Engineering, Polytechnique Montreal, Montreal, QC Canada; 2 Department of Bioengineering, McGill University, Montreal, QC Canada

**Keywords:** Surface plasmon resonance, Influenza, Sialic acid, Hemagglutinin

## Abstract

**Abstract:**

Surface plasmon resonance (SPR)-based biosensors have emerged as a powerful platform for bioprocess monitoring due to their ability to detect biointeractions in real time, without the need for labeling. Paramount for the development of a robust detection platform is the immobilization of a ligand with high specificity and affinity for the in-solution species of interest. Following the 2009 H1N1 pandemic, much effort has been made toward the development of quality control platforms for influenza A vaccine productions, many of which have employed SPR for detection. Due to the rapid antigenic drift of influenza’s principal surface protein, hemagglutinin, antibodies used for immunoassays need to be produced seasonally. The production of these antibodies represents a 6–8-week delay in immunoassay and, thus, vaccine availability. This review focuses on SPR-based assays that do not rely on anti-HA antibodies for the detection, characterization, and quantification of influenza A in bioproductions and biological samples.

**Key points:**

• *The single radial immunodiffusion assay (SRID) has been the gold standard for the quantification of influenza vaccines since 1979. Due to antigenic drift of influenza’s hemagglutinin protein, new antibody reagents for the SRID assay must be produced each year, requiring 6–8 weeks. The resulting delay in immunoassay availability is a major bottleneck in the influenza vaccine pipeline. This review highlights ligand options for the detection and quantification of influenza viruses using surface plasmon resonance biosensors.*

## Introduction

Since the 1918 influenza pandemic (H1N1 “Spanish flu”), the influenza virus has been responsible for three major pandemics, i.e., those of 1957 (H2N2 “Asian flu”), 1968 (H3N2 “Honk Kong flu”), and 2009 (H1N1 “swine flu”) (Chen et al. 2011; Kilbourne 2006). Following the most recent influenza pandemic in 2009, the World Health Organization (WHO) made a call for the improvement of vaccine production workflows. Despite major improvements on the production end, robust and rapid quality control methods are still lacking (Manceur and Kamen 2015; Thompson et al. 2013). Since 1979, the single radial immunodiffusion (SRID) assay has been the gold standard for the quantification of influenza particles, due in part to its simplicity, reproducibility, and high degree of specificity (Wood and Weir 2018). The SRID assay relies on antibodies to detect the primary antigenic protein, hemagglutinin (HA). However, due to the rapid antigenic drift of hemagglutinin’s head domain, novel anti-HA antibodies need to be produced each season once the dominant strains are selected in anticipation of the next influenza season. While vaccine production is unaffected, delays in SRID assay availability arising from lengthy antibody manufacturing process represent a major bottleneck for influenza vaccine distribution (Minor 2015). Following the 2009 H1N1 pandemic, the WHO and other regulatory health agencies identified the unavailability of SRID reagents as the primary cause of delayed vaccine distribution worldwide. In the same workshop, these agencies recommended improvements of the SRID assay, as well as the development of alternative methods for influenza vaccine quality control (Hardy et al. 2011).

Influenza refers to a group of viruses which all belong to the Orthomyxoviridae family. There exist four types of influenza viruses, termed influenza A, B, C, and D, though only influenza A and B pose a pathogenic threat to humans (Dou et al. 2018). The infectious properties of influenza viruses are governed by the activity of two major surface proteins, hemagglutinin (HA) and neuraminidase (NA), which both recognize sialic acid terminated glycostructures, expressed on the luminal surface of host epithelial tissue. Human influenza strains preferentially bind to sialic acids linked in an α2-6 “bent” conformation, whereas avian strains tend to bind to sialic acids linked in an α2-3 “straight” conformation (Gamblin and Skehel 2010; Silva et al. 2021). Following viral entry and viral RNA (vRNA) replication, the neuraminidase protein cleaves sialic acid residues to release newly replicated influenza virions from the infected host cell (Dou et al. 2018; Gamblin and Skehel 2010).

Influenza A viruses (IAV) are further subclassified into strains, which are named based on the identity of the HA and NA proteins, which in turn reflect genetic and antigenic properties. For example, the H1N1 IAV is decorated with H1 and N1 surface proteins. Other surface proteins include the matrix protein 2 (M2) (Dou et al. 2018), as well as proteins originating from the membrane of the host cell in which the Influenza virion replicated (Hutchinson et al. 2014).

Since HA is the most abundant viral membrane-bound protein and the dominant antigen, the quantity of HA is the critical quality attribute of influenza vaccine doses and is standardized around 15 µg/dose (Minor 2015). The amount of NA present for the standard 15 µg/dose of hemagglutinin can vary widely, as different influenza strains vary in their relative expression of HA and NA (Sultana et al. 2014). While influenza vaccination does produce an increase in circulating anti-NA, which reduces disease severity, anti-NA do not inhibit viral infection as effectively as anti-HA antibodies (Bright et al. 2009). In a randomly controlled trial, various commercially available inactivated influenza vaccines were found to produce significant differences in circulating anti-NA for vaccinated subjects (Couch et al. 2012). Due to its greater impact on viral immunogenicity, HA is favored as a target for the detection of influenza by regulatory health agencies.

The strategies addressed in this review will be focused on the detection of fully expressed proteins, as these are the primary immunogenic structures used in inactivated influenza vaccines (IIV), live attenuated influenza vaccines (LAIV), and recombinant influenza vaccines (Chen et al. 2020). While effective mRNA-based influenza vaccines have been demonstrated in animal models (Arevalo et al. 2022), these will not be addressed in this review.

The SRID assay has been long favored as it is simple to perform, requires no specialized equipment and quantification by SRID has a good correlation with antigenicity. However, this method is time-consuming and is not suitable for quantification at all stages of or the production, purification, and packaging pipeline, as some cell-lysate contaminants or vaccine adjuvants can interfere with HA migration through the agarose matrix (Thompson et al. 2013). The critical disadvantage of SRID is its reliance on novel anti-HA antibodies, which can only be produced once the dominant seasonal strain (or pandemic strain) has been identified. This disadvantage is shared by other antibody-dependent analytical assays, such as the Western blot (Thompson et al. 2015) or enzyme-linked immunosorbent assays (ELISA) (Hashem et al. 2013). This review addresses the various non-antibody ligands which have been used to detect influenza using surface plasmon resonance (SPR) biosensors. These ligand choices would be equally valuable for detection using other optical (ex. biolayer interferometry analysis) (Carvalho et al. 2017; Petersen 2017) or piezoelectric (ex. quartz crystal microbalance) (Lim et al. 2020) biosensors which employ immobilized ligands for analyte detection.

## SPR biosensors

Surface plasmon resonance is a powerful tool for the detection of biointeractions in real time, without the need for a molecular label. Most popular SPR biosensor designs rely on Kretschmann geometry, developed in the late 1960s (Kretschmann and Raether 1968).

**Fig. 1:**
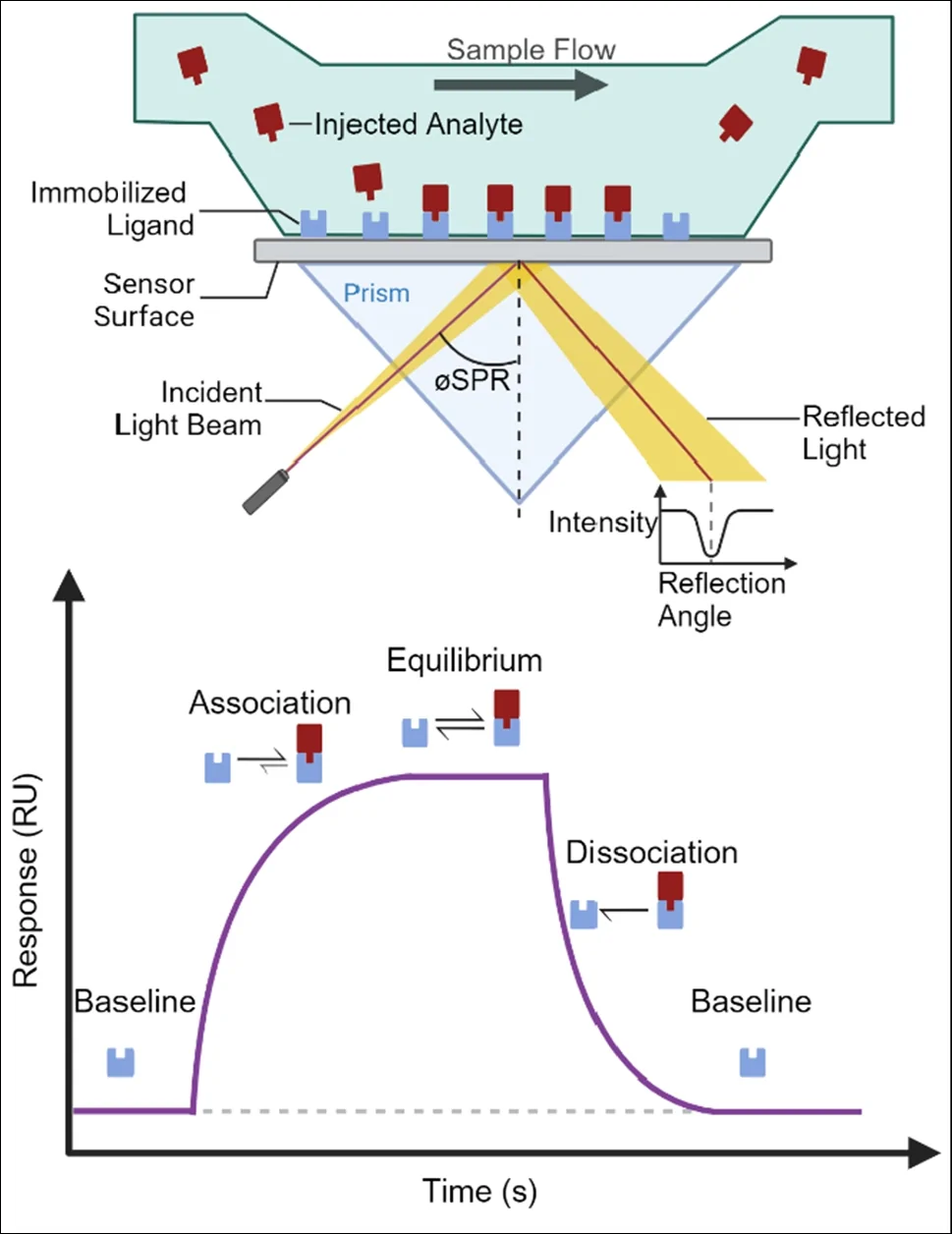
Top schematic representation of the flow cell, prism, and light emission and detection system in the Kretschmann configuration. Bottom: phases of a SPR sensorgram, overlaid with the ligand-analyte interactions taking place

In the Kretschmann configuration, a sensor chip, consisting of a thin film of gold or silver, sits atop a prism. On the opposite side, the sensor surface, a dielectric medium provides an adequate milieu for biointeractions and also contributes to detection due to its specific optical properties (Maurya and Prajapati 2016). During detection, an incident beam of light scans across the metal film from beneath the sensor surface and excites electrons from the metal into oscillation clouds known as plasmons. Due to plasmon generation, the light reflected off the sensor chip is of lower intensity. The angle of the reflected light beam at which the peak intensity dip occurs is known as the SPR angle. The SPR angle is a function of the refractive index of the dielectric medium above the surface and therefore of the concentration of matter in proximity (300 nm) of the surface (Fig. 1, top).

Onto the surface of an SPR sensor chip, the ligand is covalently immobilized or reversibly captured. During the association phase, the analyte is injected into the flow cell. As analytes interact with ligands and mass accumulates at the surface, the refractive index of the dielectric medium changes, resulting in a shift in the SPR angle, expressed in arbitrary resonance units (RU). Plotting RU versus time provides an insight on kinetics of biomolecular interactions between the surface-bound ligand and the injected analyte (Damborsky et al. 2016; Gaudreault et al. 2021; Helmerhorst et al. 2012; H. H. Nguyen et al. 2015) (Fig. 1, bottom). Alternative signal generation methods and sensor chip designs, intended to increase sensitivity, are beyond the scope of this review (Puiu and Bala 2016).

**Fig. 2:**
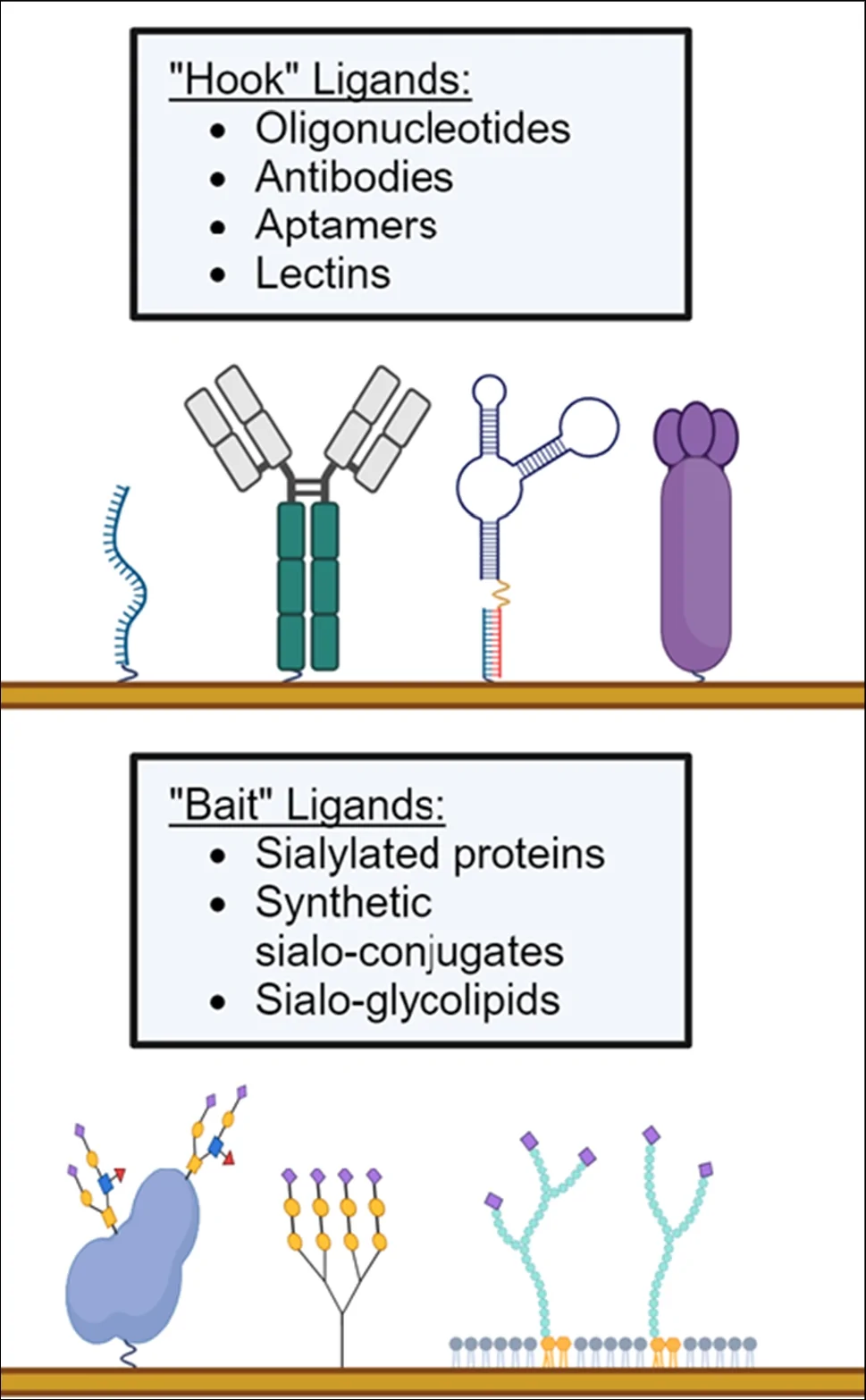
Summary of “hook” and “bait” ligands suitable for the detection of influenza using SPR biosensors

Beyond these basic principles, each SPR experiment is designed to satisfy the experimenter’s objectives. For any given ligand-analyte pair, the assay configuration, ligand immobilization method, ligand density, analyte flow rate, analysis temperature, and reference surface design can be modified to accomplish the desired objectives. This review will focus specifically on the selection of SPR ligands for the detection of influenza. The ligands presented can be separated into two broad classes. “Hook” ligands bind to influenza via the ligand’s binding domain (Fig. 2, left), while “bait” ligands capture influenza by presenting a target for the viral surface protein hemagglutinin, mimicking the infection mechanism (Fig. 2, right).

The primary advantage of SPR biosensors is their autonomy and high throughput, thanks to the implementation of efficient liquid handling systems in most commercial SPR biosensors. Detection by SPR occurs in real time as the biointeraction is occurring, resulting in very short analysis times compared to methods like SRID (Khurana et al. 2014). While SPR biosensors can be used for analyte detection in crude samples, this approach requires a more careful SPR surface design (i.e., non-fouling matrix, highly specific ligand) to prevent the generation of non-specific signals (Couture et al. 2013). Compared to microplate (ELISA) or gel-based (SRID, Western Blot) methods, SPR analysis is very expensive due to the high cost of the biosensor and of the disposable sensor chips.

## Oligonucleotides

Oligonucleotide ligands detect vRNA from lysed viruses and are favored for diagnostic or screening purposes. To obtain strong signals, the concentration of vRNA is amplified via reverse-transcription polymerase chain reaction (RT-PCR), and the amplified vRNA is subsequently injected over a surface bearing a complementary oligonucleotide ligand. The main advantage of oligonucleotide ligands is their high specificity for the complementary vRNA sequence, which enables the detection of vRNA in complex biological samples (Shi et al. 2015).

**Fig. 3:**
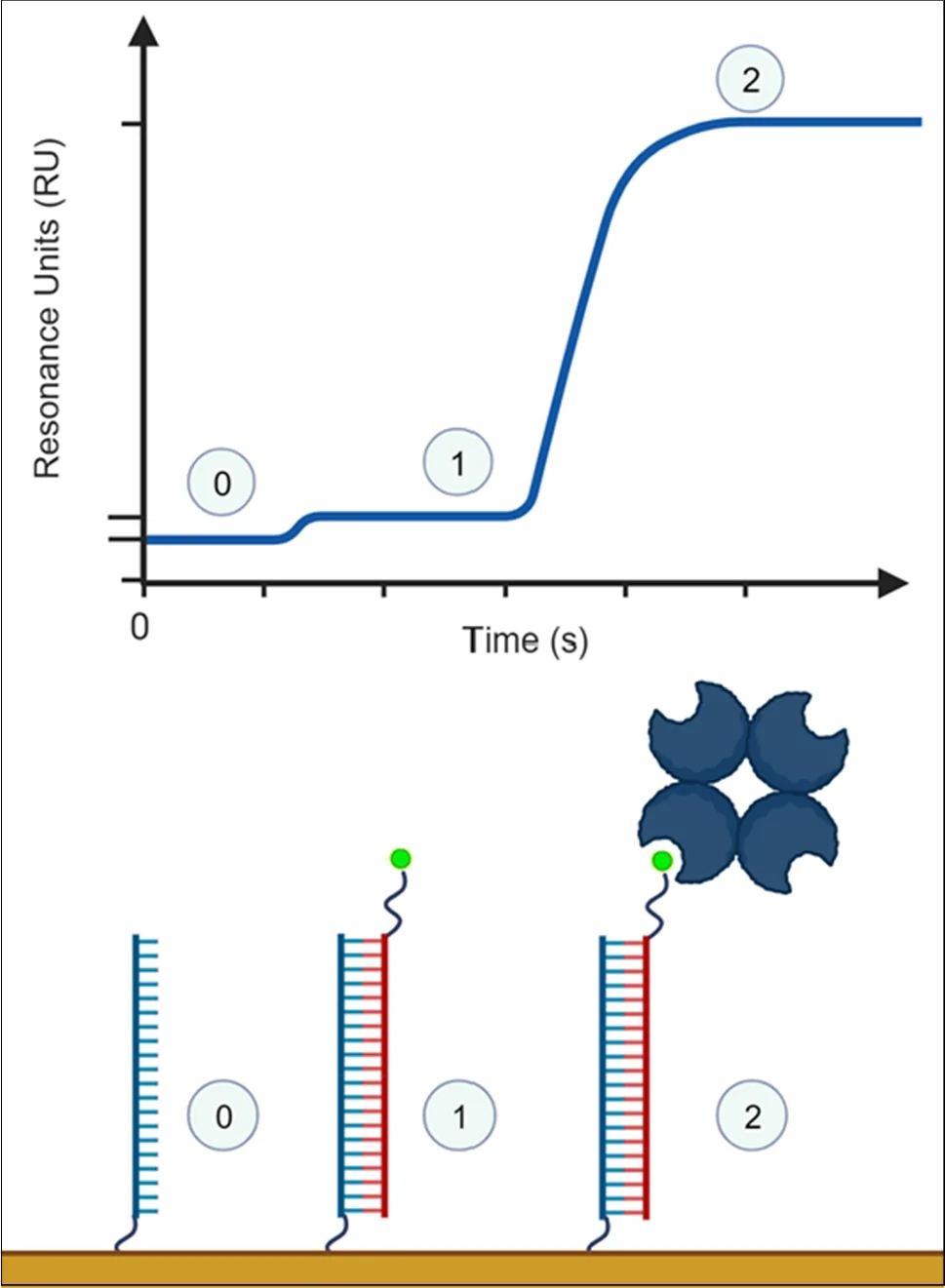
Schematic representation of signal amplification using a biotin tag to recruit a high molecular weight protein (e.g., streptavidin). (0) The baseline, where only the immobilized ligand is present at the surface. (1) Binding of the low molecular weight biotin-tagged analyte. Since the SPR signal is proportional to mass accumulated at the surface, the resulting signal is weak. (2) Binding of a high molecular weight protein (streptavidin) to the biotin tag, resulting in an analyte-specific signal amplification

However, vRNA analytes have a relatively low molecular weight and would therefore produce weak signals in SPR biosensors as the SPR signal is proportional to the accumulation of mass at the sensor surface (Gaudreault et al. 2021). To overcome this limitation, one approach is the inclusion of a tag, like biotin, to the terminus of the amplified vRNA product. Following the initial vRNA-RNA interaction, a molecule with specificity toward the tag, streptavidin in this example, can be injected to amplify the specific signal (Shi et al. 2015) (Fig. 3).

Another disadvantage of vRNA-based detection is that only live attenuated influenza vaccines (LAIV) containing complete vRNA sequences can be analyzed. Inactivated influenza vaccines (IIV) contain damaged vRNA, whose molecular weight as well as specificity for the immobilized oligonucleotide would be highly inconsistent, depending on the inactivation strategy employed. Recombinant vaccines are devoid of complete vRNA strands (Herrera-Rodriguez et al. 2019) and thus are unsuitable for this oligonucleotide-based approach. Due to the need for vRNA amplification, this method is not suitable for in-line detection.

## Anti-HA2 antibodies

**Fig. 4:**
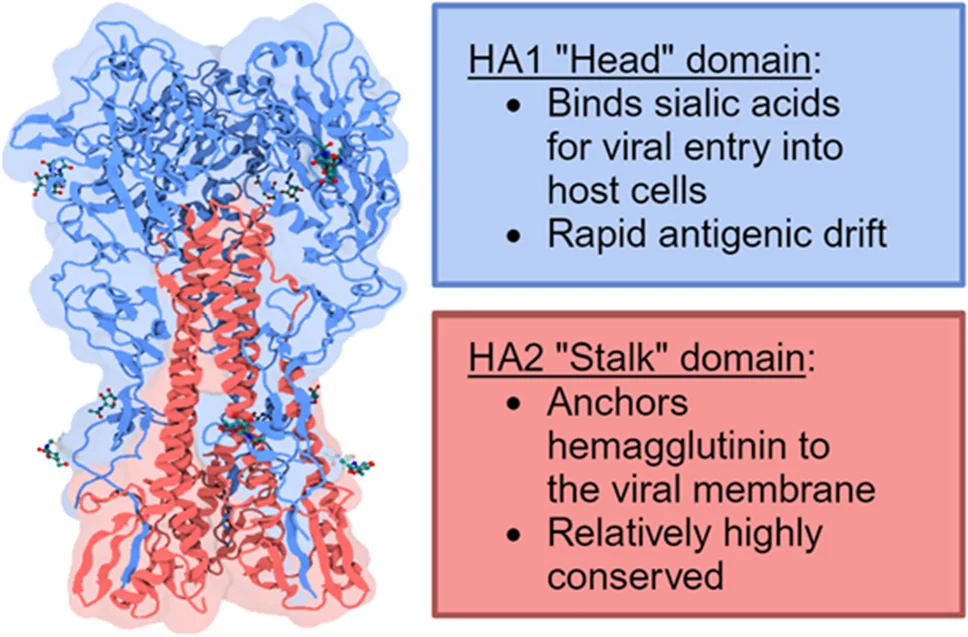
3D conformation of the homo-trimeric hemagglutinin protein from influenza A H1N1. In blue, the HA1 “head” domain, susceptible to rapid antigenic drift and the target for immunodetection in the traditional SRID assay. In red, the HA2 “stalk” domain, whose structure is less susceptible to antigenic drift. Protein Data Base ID: 3HTO (Lin et al. 2009)

While the globular HA1 “head” domain exhibits rapid antigenic drift, the HA2 “stalk” domain, which anchors HA to the viral envelope, is more conserved over time and across influenza A subtypes (Fig. 4). These conserved regions are attractive targets for “universal” anti-HA antibodies, resistant to antigenic drift and shift. Anti-HA2 antibodies have been proposed as treatments for severe influenza infections, as they are thought to inhibit viral attachment, fusion to the lysosome, and release of newly replicated virions. In the interest of this review, anti-HA2 antibodies would also be valuable for quantification, as they would not require seasonal updates.

Anti-stalk domain antibodies are usually not produced following an immunization with an IIV, as HA1 is more immunogenic (Krammer 2016). The most conserved region, and thus most attractive target for a universal anti-HA2 antibody, is the fusion peptide. This sequence is found at the N-terminus of the HA2 domain and is highly conserved across all influenza A subtypes (Gerhard et al. 2006). Production of universal antibodies specific this structure was achieved via animal immunization using synthetic peptides which have the same amino acid sequence as the fusion peptide. These universal stalk-reactive antibodies effectively detect recombinant HA and final vaccine formulation from multiple IAV strains, though signal strength varied from strain to strain (Chun et al. 2008; Manceur et al. 2017). Though quantification was demonstrated with ELISA (Chun et al. 2008) and slot-blot assays (Manceur et al. 2017), quantification was not demonstrated using SPR biosensors. However, antibodies have been widely used as SPR ligands.

In the works presented above, detection using these antibodies was achieved following denaturation with urea to expose the fusion peptide domain, which is otherwise obstructed by the HA1 “head” domain. Since pretreatment is necessary, these antibodies would be unsuitable for in-line monitoring. For the same reason, they would not provide information on the concentration of conformationally active HA, which is relevant for immunogenicity.

## Aptamers

Aptamers are short, single-stranded oligonucleotide sequences that adopt 3D conformations to interact with biomolecules. Aptamers display specificity and affinity for their biological partners on par with antibodies. The conventional process of aptamer design is known as systematic enrichment of ligands by exponential enrichment (SELEX). Herein, an initial oligonucleotide pool (IOP) consisting of nearly every nucleotide sequence of a given length (30–80 bp) is incubated with a solid-phase target analyte. Due to competition for the analyte protein, the aptamers with the greatest affinity for the analyte will remain bound, while those with weaker or null interactions will be washed away. Following an elution step, the aptamers with high affinity are amplified by PCR. The cycle of competitive binding and amplification continues only few aptamers remain, and whose affinities for the analyte have reached the nM-pM range (Lakhin et al. 2013).

SELEX-derived aptamers have been employed in numerous SPR assays for the detection of influenza A (Bai et al. 2012; Gopinath and Kumar 2013; Gopinath et al. 2006a; Misono and Kumar 2005; Nguyen et al. 2016; Wang et al. 2013) and B (Gopinath et al. 2006b). HA-specific aptamers have displayed superior specificity to antibodies when it comes to differentiating between IAV subtypes (Wang et al. 2013) and have been used for the detection of influenza virions in complex biological samples (Bai et al. 2012). Their binding affinity for HA is comparable with antibodies and can be further increased through chemical modifications. In one work, aptamers constructed using 2′fluoropyrimidine in place of pyrimidine base pairs displayed a thermodynamic dissociation constant (*K*_D_) on the femtomolar scale (K_D_ ~ 10^−14^ M) (Gopinath and Kumar 2013). Using high affinity ligands is beneficial as it lowers the limit of detection for SPR assays (Schuck and Zhao 2010).

One of the biggest advantages of aptamers is their specificity, which can be enhanced via negative SELEX or counter SELEX. In this approach, the aptamers are first incubated with the non-target contaminants that may be present in the final sample (ex. host-cell proteins). The aptamers which display no specificity for these non-targets are then collected and then used for a conventional SELEX-based aptamer selection. The result is a highly selective aptamer that can distinguish between different IAV subtypes (V. T. Nguyen et al. 2016) or even within the same subtype from different seasons (Gopinath and Kumar 2013; Gopinath et al. 2006a). Aptamers designed in this fashion are good candidates for detection in crude mediums, as they can be intentionally designed to show no specificity for host-cell proteins.

One disadvantage of aptamers is the presence of amine functional groups in guanine, adenine, and cytosine, making them unsuitable for amine coupling via carbodiimide chemistry. Aptamers require capture tags, which are commonly biotin (Bai et al. 2012; Nguyen et al. 2016; Wang et al. 2013), or poly-adenine tail (Gopinath and Kumar 2013; Gopinath et al. 2006a, b; Misono and Kumar 2005). These tags are used for capture of aptamers to surfaces functionalized with streptavidin or poly-thymine tails, respectively.

## Lectins

Lectins are non-catalytic sugar-binding proteins which play a role in cellular signaling and adhesion via interactions between sugar structures and the lectin’s carbohydrate recognition domain (CRD) (Van Breedam et al. 2014). Since HA is a glycoprotein (An et al. 2013), lectins are attractive candidates for detection in SPR biosensors.

The main advantage of lectins is their indifference toward the antigenic drift of the HA head domain. However, there is significant variability in viral glycosylation patterns. Since protein glycosylation is achieved via the combined actions of host-cell glycotransferases and glycosidases (Schjoldager et al. 2020), the glycosylation patterns of biomanufactured HA will depend largely on the cell lines and culture conditions in which the viruses replicate (An et al. 2013). For reliable detection using lectins, strict control of culture conditions and medium components is required. Host-cell gene modification or addition of glycosylation inhibitors could be used to further control glycosylation and improve consistency from batch to batch (Butler and Spearman 2014). Control of glycosylation in vaccine production could be a valuable research avenue, as HA glycosylation has been demonstrated to play an important role in protein folding, stability, pathogenicity, and affinity for sialic acid decorated glycostructures. Certain HA glycosylation patterns are also associated with increased innate immune response from an infected host (York et al. 2019) and must be entirely avoided.

Due to the reliance on host-cell machinery for HA glycosylation, identical influenza strains can bind differently to lectin surfaces depending on the production platform (Opitz et al. 2008). Due to interlot variability of HA glycosylation, as well the presence of multiple glycans within the same glycostructure, multiple lectin candidates can viably act as ligands for the same influenza batch (Mandenius et al. 2008). Tight control of glycosylation upstream is critical to the reliability of lectin biosensors for HA detection.

Since SPR biosensors only detect the accumulation of mass near the surface and the specificity of lectins toward sugars as opposed to protein structures, lectin biosensors would be unable to distinguish between HA and similarly glycosylated host-cell proteins. Lectins would be better suited for HA glycosylation analysis in purified samples, rather than HA quantification in crude cell lysates.

## Sialic acid decorated surfaces

The most common ligand choice for the detection of influenza is a sialic acid decorated surface. In this approach, sialic acid residues serve as “bait” for viral HA proteins and present a surface that mimics the natural infection mechanism.

The affinity between HA and a single sialic acid residue is in the mM-µM range. Due to the trivalency of HA, as well as high density of HA on the viral membrane (Luo 2012), influenza virions display multivalent binding, increasing the affinity to the low nM range (Kosik and Yewdell 2019). Additionally, IAVs display a preference for tissues which express a high density of sialic acid terminated glycostructures such as the respiratory lumen in humans and swine or the enteric lumen in avian species (Gamblin and Skehel 2010). This “cluster effect,” wherein multivalency increases apparent affinity of lectin-sugar interactions, is a feature of most animal, plant, and viral lectins (Van Breedam et al. 2014; Varki et al. 2009a). To exploit this cluster effect, sialic acid tends to be immobilized at a high density on SPR surfaces. One major disadvantage is that sialic acid decorated surfaces are susceptible to degradation by neuraminidase, which cleaves sialic acids from host surfaces to enable release of the newly replicated influenza virion.

Occasionally, avian influenza strains acquire specificity for α2-6 linked sialic acid residues. The sudden shift introduces highly pathogenic influenza strains to the human immune system (Chen et al. 2011; Stencel-Baerenwald et al. 2014). Sialic acid terminated ligands are effective ligands for the detection of avian influenza strains that have acquired interspecies infectivity, as was the case for the 2009 H1N1 “swine flu” pandemic (Suenaga et al. 2012). A number of approaches toward sialic acid decorated surfaces have been reported.

## Sialylated glycoproteins

Naturally occurring sialylated glycoproteins are effective bait ligands for influenza detection. Since sialic acid terminated glycans fall under the category of “complex type” glycans, these glycoproteins are only expressed in higher order organisms, such as vertebrates (Varki et al. 2009b). Complex-type glycans are usually branched. Branching, in combination with the potential for multiple glycosylation sites on a single protein, contributes toward the cluster effect.

In one work, sialylated human serum glycoprotein protein α1-AGP, bearing 5 potential N-glycosylation sites, was successfully used to detect HA from a human influenza strain (Mandenius et al. 2008). In another work, bovine serum fetuin, a glycoprotein bearing both α2-3 and α2-6 linked sialic acid residues, was used to quantify the HA present in a vaccine lot. More impressively, this same surface design was selective enough to detect H1N1 in a clarified cell supernatant. In the same work, the authors reported no binding between immobilized fetuin and monomeric recombinant HA, likely due to the absence of avidity binding. An adequate reference surface was prepared with asialofetuin, to subtract the signals arising from non-specific interactions (L. Durous et al. 2019). The main advantage of sialylated glycoproteins is their commercial availability and their indifference to antigenic drift and shift. However, these naturally occurring ligands are less well controlled, with respect to their glycosylation, compared to synthetic alternatives.

## Synthetic glycoconjugates

Synthetic sialylated ligands are highly versatile tools, as they can be precisely designed to accommodate specific experimental requirements. One approach to the construction of synthetic glycoconjugate ligands is the conjugation of a glycan to a carrier protein (Mandenius et al. 2008), which can be covalently coupled to an SPR surface. Other groups opt for entirely synthetic glycans constructed onto a PAA (polyacrylamide) backbone which carries a biotin tag for immobilization to streptavidin (Khurana et al. 2014; Suenaga et al. 2012).

The advantage of synthetic glycans compared to naturally occurring glycoproteins is that their structure can be customized depending on the specific parameters of the assay. For example, the apparent affinity of the HA-glycan interaction can be fine-tuned by changing the conjugation ratio of the glycan on the carrier protein(Mandenius et al. 2008). While high affinity ligands have a lower limit of detection, medium–low affinity ligands are more suitable for in-line applications, as these surfaces would not require surface regeneration steps (Mandenius et al. 2008).

The synthetic approach also provides the ability to control the attachment orientation of the terminal sialic acid, for the detection of avian or human influenza strains. One such strategy involved selective use of either α2-3-sialyltransferase or α2-6-sialyltransferase to control the linkage of sialic acid to the subterminal galactose residue (Narla and Sun 2012). Alternatively, linkages for which no influenza strains display specificity, like α2-8 attachment, can be produced and used as reference ligands to construct “blank slide” control surfaces (Khalenkov et al. 2023).

## Sialoglycolipids

To further emulate physiological conditions, sialoglycan decorated lipid monolayers can be immobilized to hydrophobic sensor chips. In one work, naturally sialylated bovine brain lipid neomembranes were immobilized to a SPR sensor chip. While the surface successfully interacted with injected IAV, the sialoglycolipids were highly susceptible to degradation neuraminidase, giving rise to baseline drifts over the course of repeated injection cycles (Critchley and Dimmock 2004).

## Perspectives/conclusion

The development of novel, antibody-independent influenza quantification assays is critical for the improvement of the vaccine distribution pipeline and will reduce the time to vaccine availability by weeks in a pandemic scenario. Among the alternative strategies proposed by regulatory health agencies following the most recent influenza pandemic, SPR biosensors have already been demonstrated to be powerful tools for in-line and off-line monitoring of biomanufacturing. To adequately replace SRID, SPR assays for influenza detection will require highly selective, high affinity ligands immobilized to the sensor surface. Crucially, these ligands must either be rapidly produced once a dominant influenza strain has been identified or must target structures other than the HA1 “head” domain and therefore be independent of influenza’s rapid antigenic drift. For in-line applications, surface designs should also consider reduction of non-specific binding from contaminants present in complex solutions such as cell culture medium. Additionally, SPR quality control assays should be aimed at multiple stages of the vaccine production pipeline, from crude in-line samples to final vaccine formulations in the presence of adjuvants.

Sialic acid terminated “bait” ligands, particularly synthetic ones, are the most versatile option for influenza detection. The primary advantages of these ligands are unaffected by antigenic drift and shift of HA and can thus be prepared in advance of dominant strain identification. By mimicking the conditions necessary for influenza infection, these ligands selectively recognize HA which expresses the correct conformation and therefore which has immunogenic potential. It is also suitable for use in crude samples, though additional measures should be taken to reduce non-specific binding. Synthetic ligands or glycoconjugates are favorable to naturally occurring sialylated glycoproteins, as they provide more avenues for optimization via affinity tuning (Mandenius et al. 2008).

To date, SRID remains the gold standard method for influenza quantification. Much of the effort to replace SRID was motivated by the 2009 pandemic. Recently, few novel approaches have emerged. Much of the efforts regarding influenza quality control by SPR involve the integration of these biosensors in-line (Laurent Durous et al. 2023) or for pharmacokinetic evaluation of influenza drugs which inhibit the actions of viral proteins (Liu et al. 2023; Ma et al. 2024). The influenza vaccine distribution pipeline will also benefit from lessons learned during the recent COVID-19 pandemic (Buchy et al. 2021; Scala et al. 2023).

SPR biosensors have been used in the development and monitoring of vaccines and drugs which protect against other viral infections, including HIV, malaria, SARS, hepatitis C (Hearty et al. 2010), and more recently for SARS-CoV-2 (Szunerits et al. 2022). Many of the reported ligand options reported here can be applied in these other viral vaccine pipelines, though the specific approaches are outside the scope of this review.
